# Prophylactic Intra-Peritoneal Drainage After Pancreatic Resection: An Updated Meta-Analysis

**DOI:** 10.3389/fonc.2021.658829

**Published:** 2021-05-20

**Authors:** Xinxin Liu, Kai Chen, Xiangyu Chu, Guangnian Liu, Yinmo Yang, Xiaodong Tian

**Affiliations:** Department of General Surgery, Peking University First Hospital, Beijing, China

**Keywords:** intra-peritoneal drainage, pancreatic resection, pancreaticoduodenectomy, distal pancreatectomy, meta-analysis

## Abstract

**Introduction:**

Prophylactic intra-peritoneal drainage has been considered to be an effective measure to reduce postoperative complications after pancreatectomy. However, routinely placed drainage during abdominal surgery may be unnecessary or even harmful to some patients, due to the possibility of increasing complications. And there is still controversy about the prophylactic intra-peritoneal drainage after pancreatectomy. This meta-analysis aimed to analyze the incidence of complications after either pancreaticoduodenectomy (PD) or distal pancreatectomy (DP) in the drain group and no-drain group.

**Methods:**

Data were retrieved from four electronic databases PubMed, EMBASE, the Cochrane Library and Web of Science up to December 2020, including the outcomes of individual treatment after PD and DP, mortality, morbidity, clinically relevant postoperative pancreatic fistula (CR-POPF), bile leak, wound infection, postoperative hemorrhage, delayed gastric emptying (DGE), intra-abdominal abscess, reoperation, intervened radiology (IR), and readmission. Cochrane Collaboration Handbook and the criteria of the Newcastle-Ottawa scale were used to assess the quality of studies included.

**Results:**

We included 15 studies after strict screening. 13 studies with 16,648 patients were analyzed to assess the effect of drain placement on patients with different surgery procedures, and 4 studies with 6,990 patients were analyzed to assess the effect of drain placement on patients with different fistula risk. For patients undergoing PD, the drain group had lower mortality but higher rate of CR-POPF than the no-drain group. For patients undergoing DP, the drain group had higher rates of CR-POPF, wound infection and readmission. There were no significant differences in bile leak, hemorrhage, DGE, intra-abdominal abscess, and IR in either overall or each subgroup. For Low-risk subgroup, the rates of hemorrhage, DGE and morbidity were higher after drainage. For High-risk subgroup, the rate of hemorrhage was higher while the rates of reoperation and morbidity were lower in the drain group.

**Conclusions:**

Intraperitoneal drainage may benefit some patients undergoing PD, especially those with high pancreatic fistula risk. For DP, current evidences suggest that routine drainage might not benefit patients, but no clear conclusions can be drawn because of the study limitations.

## Introduction

Pancreatic resection is an essential treatment for malignancy/benign lesions of the pancreas, and includes two main types in the procedure: pancreaticoduodenectomy (PD) and distal pancreatectomy (DP) (1, 2). Despite the development of surgical techniques and experience, the incidence of complications after pancreatectomy is still as high as 30%-50% (3–5). Pancreatic fistula is one of the major complications and a major factor related to morbidity and mortality in patients undergoing pancreatic resection (5–7). Traditionally, routine intra-peritoneal drainage after pancreatectomy was considered to be an effective measure to reduce postoperative complications (8, 9). However, some studies have shown that prophylactic placement of abdominal drainage could not reduce and even increase the incidence of postoperative complications (10, 11). So there is still controversy about the prophylactic intra-peritoneal drainage after pancreatectomy.

So far there have been several randomized controlled trials (RCTs) or nonrandomized controlled trials (nRCTs) which suggested that routine intra-peritoneal drainage failed to reduce postoperative complications or even increased the frequency and severity of complications (10–16). In 2001, Conlon et al. (10) conducted the first RCT and showed that drainage could not reduce the number of death or complication after pancreatic resection, which was consistent with the report of Witzigmann et al. (11) on PD. These studies indicated that prophylactic drainage should not be used as a standard pattern after pancreatic surgery. However, Van Buren et al. (17) reported that drainage in all cases of PD significantly reduced the frequency and severity of complications. In their later study on a series of 344 patients with DP, no significant differences were found between drain and no drain groups in terms of relevant postoperative complication except intra-abdominal fluid collection (18). The inconsistent results in different studies may be due to small sample size, lack of discrimination between PD and DP, and no stratification for patients with different fistula risk score (FRS).

Several systematic and meta reviews on this controversial topic have been published in recent years (19–23). Most reviews showed that placement of drainage can reduce the rate of mortality, but may increase the incidence of complications. However, there are some limitations in previous meta-analyses. First, the different types of surgery such as PD and DP were not discussed separately, which could have a confounding effect on the significance for clinical practice (9). The incidence of fistula is generally higher in DP than in PD, while the severity of pancreatic fistula in DP is lower (9, 24–26). Moreover, the incidences of other postoperative complications are incompatible. Second, there is no stratification of patients with different FRS. The prophylactic drainage may be more suitable for patients with high pancreatic fistula risk due to high incidence of postoperative complications (9). Therefore, this study aimed to assess the effect of intraperitoneal drains in patients undergoing pancreatic resection by excluding the bias shown above. We performed updated meta-analysis based on currently available data on the incidence of complications after either PD or DP in the drain group and the no-drain group.

## Materials and Methods

### Literature Retrieval

The following medical terms were used to search all the literatures in electronic databases PubMed, EMBASE, the Cochrane Library and Web of Science up to December 2020: “pancreaticoduodenectomy”, “pancreatectomy”, “pancreatic disease/surgery”, “pancreatoduodenectomy”, “distal pancreatectomy”, “pancreatic resection”, “pancreas”, “pancreas*”, “drainage”, “drain”, “drain*”, “suction”, “suction*”, and “negative-pressure wound therapy”, which were used in combination with Boolean operators AND or OR. Furthermore, the reference lists of relevant retrieved articles were screened manually to identify eligible studies. All prospective and retrospective studies with human pancreatic resections for the comparison of the effect of drains versus no drains were included.

### Inclusion and Exclusion Criteria

For inclusion in the meta-analysis to be eligible, the following inclusion and exclusion criteria were used as the guidelines in the assessment of literatures by two reviewers independently. Disagreements were resolved by the third reviewers who is an expert in this field. Inclusion criteria were: (1) the types of pancreatic surgery were PD or DP; (2) compared complications of intraperitoneal drain to no drain after PD or DP; (3) prospective or retrospective studies; (4) provided data on any complications; and that (5) the full text of original article in English could been accessed. Exclusion criteria were as follows: (1) the types of surgery were not clear, or patients did not receive the treatment of resection; (2) other kinds of drainage instead of abdominal drainage, such as biliary drainage and nasogastric drainage; (3) review, case report, abstracts, editorials, letters to the editor, and conference references; (4) no access to full-text or primary data; and (5) repeated publications.

### Data Extraction and Quality Assessment

Data were extracted from the included studies including authors, year of publication, country, inclusion year in studies, number of patients, type of surgery, age, gender, malignant and benign diseases, body mass index (BMI), and outcomes of individual treatment after PD or DP mortality, morbidity, CR-POPF, bile leak, wound infection, postoperative hemorrhage, delayed gastric emptying (DGE), intra-abdominal abscess, reoperation, intervened radiology (IR), and re-admission. The quality of included studies was assessed based on Cochrane Collaboration Handbook (27) (for RCTs) and the criteria of the Newcastle-Ottawa scale (28) (for non-RCTs).

### Outcome Definition and Subgroup

Mortality was defined as death within 30 days after pancreatic resection. Morbidity indicated overall complications. CR-POPF, DGE and postoperative hemorrhage were defined based on International Study Group on Pancreatic Fistula (ISGPF) definition (29). The data for other outcomes were collected according to the respective definitions in the included studies. The different types of surgery were divided into two subgroups. According to FRS, low risk and high risk for PD were analyzed as different subgroup (30). The rank of fistula risk was defined as the criteria in the original studies, low risk included negligible/low fistula risk and high risk included moderate/high fistula risk.

### Statistical Analysis

Review Manager (RevMan) version 5.4 (The Cochrane Collaboration, 2020) was used for meta-analysis. For dichotomous outcomes, pooled odds ratios (ORs) with a 95% confidence interval (95%CI) were calculated in model of the fixed effects. I^2^ index was used to evaluate the heterogeneity. I^2^ value > 50% indicated heterogeneity, and random effects models were used to replace fixed effects models. The effect of individual studies on the overall results was analyzed by removing relative studies for sensitivity analysis. Potential publication bias was assessed by constructing funnel plots asymmetry according to the recommendation of the Cochrane handbook for systematic reviews (27).

## Results

### Characteristic of Included Studies

**Figure 1:**
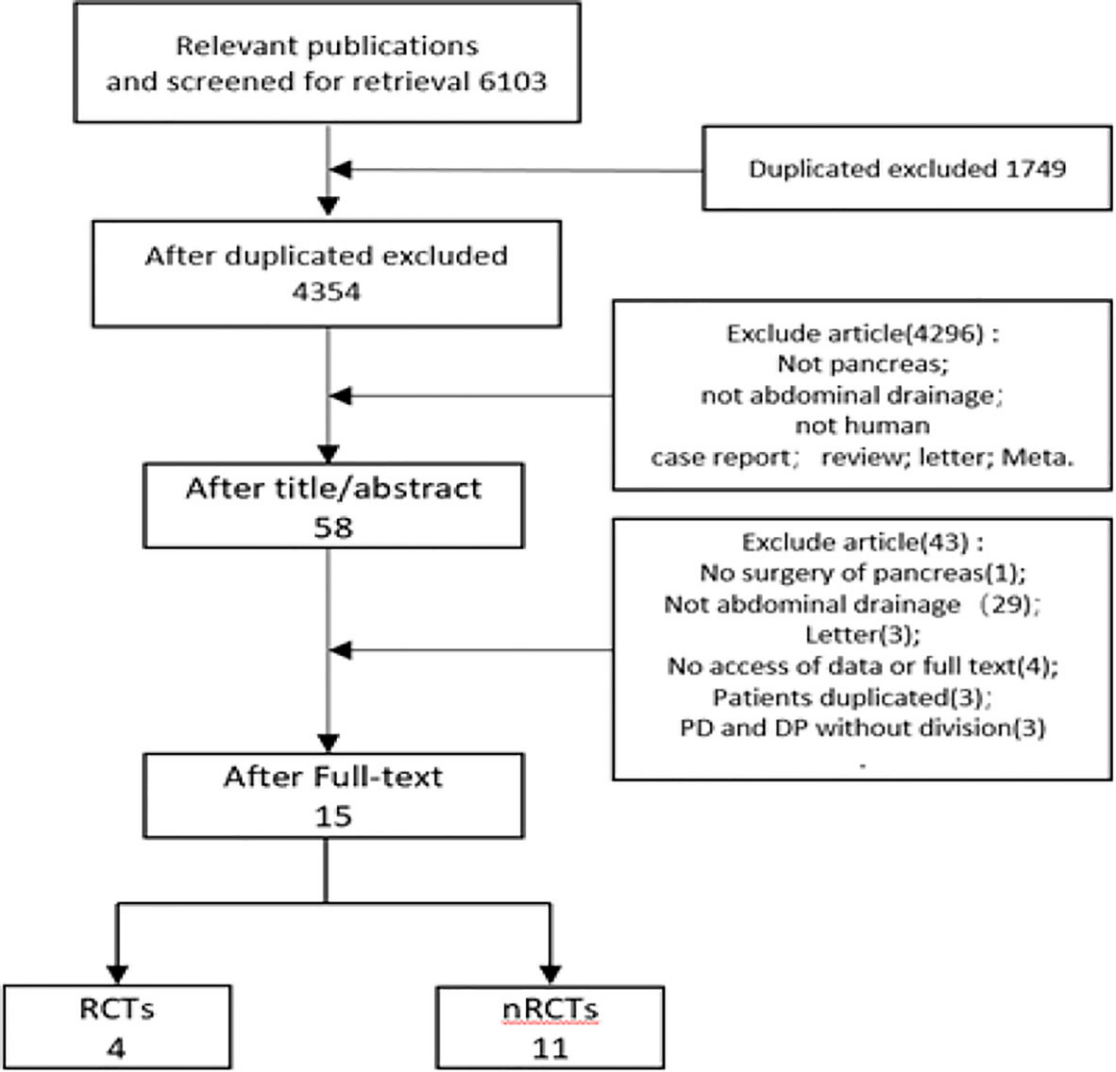
Flow chart of literature search process.

**Figure 2:**
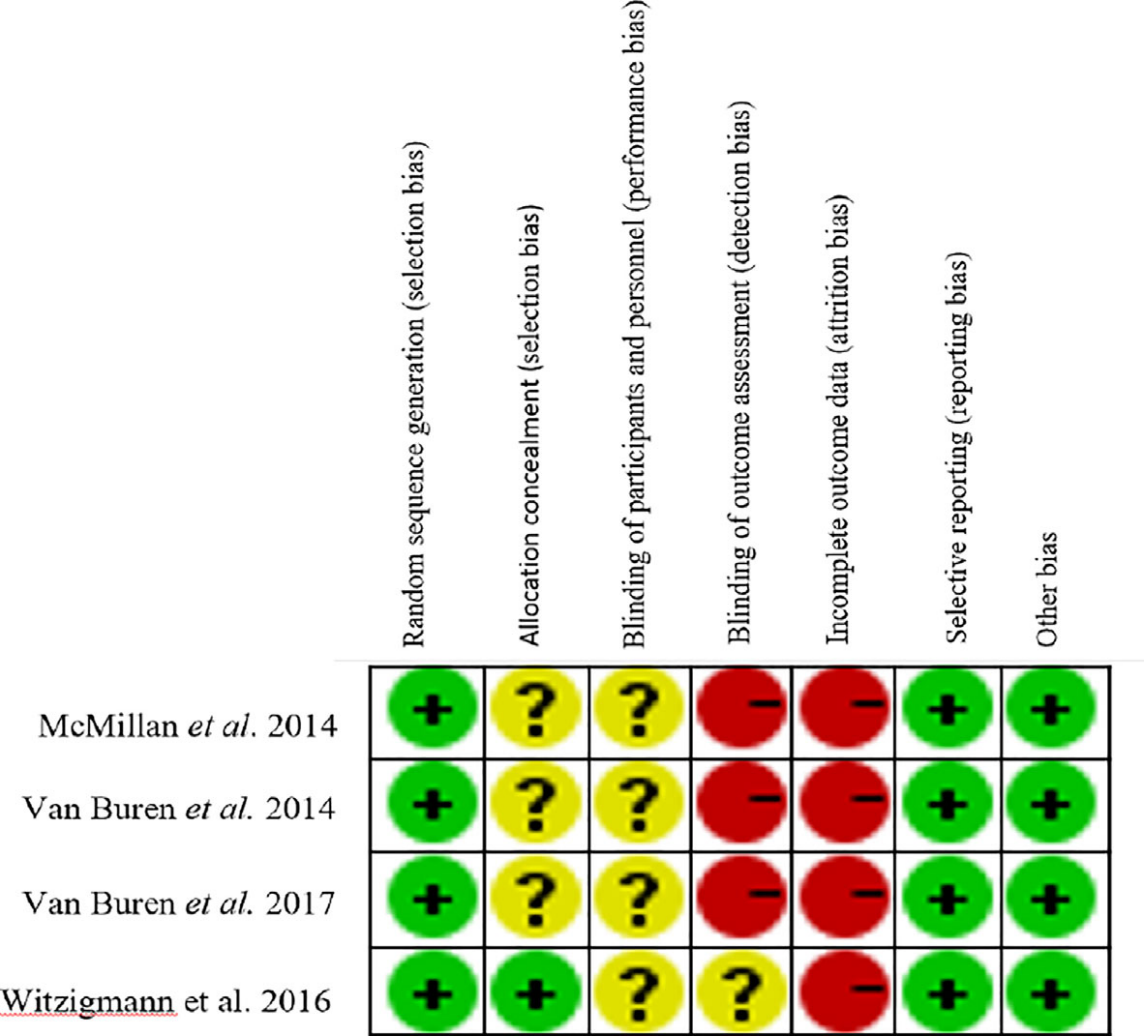
The assessment of the quality of RCTs.

Total 6,103 papers were retrieved following the designed search strategy. We excluded 1,749 duplicated papers. After screening title and abstract manually by two reviewers, 58 potentially eligible studies were selected, 15 of which were finally included after full-text scanning (11, 12, 14–18, 31–38). In this meta-analysis, five studies were prospective and ten studies were retrospective. **Figure 1** showed a flowchart of literature search process. The characteristics of the included articles were shown in **Table 1**, and the quality of the included RCTs were shown in **Figure 2**.

**Table 1:** Characteristics of studies included.

Study	Type of Study	Country	Inclusion yr(from until)	No. total	Surgery	Subgroup	Age (yr, median)	Male/Female	No.	Phatology (malignancy/benign)	BMI	NOS
nRCT studies												
Addison et al. (37)	retrospective	USA	2015-2016	7583	PD	drain	64.7±11.7	3612/3054	6666	5461/1205	27.4±6.0	8
						no drain	65.3±1 1.3	488/429	917	774/143	26.6±5.4	
Behrman et al. (34)	retrospective	USA	2011-2012	761	DP	drain	57	49/67	116	81/35	–	8
						no drain	59	55/61	116	72/44	–	
Correa-Gallego et al. (15)	retrospective	USA	2006-2011	1122	PD,DP	drain	65±13	548/574	553	458/664	–	8
						no drain			569			
Heslin et al. (12)	retrospective	USA	1994-1996	89	PD	drain	65±2	32/19	Sl	47/4	–	5
						no drain	65±2	18/20	38	31/7	–	
Kunstman et al. (35)	retrospective	USA	2003-2007	106	PD	drain	63.3±10	31/22	53	361/7	–	7
						no drain	62.2±12.4	20/33	53	39/14	–	
Lim et al. (33)	retrospective	France	2009-2011	54	PD	drain	62 (40-76)	8/19	27	20/7	23.5 (18-39)	6
						no drain	62 (38-78)	8/19	27	20/7	23 (17-39)	
McMillan et al. (36)	prospective	Italy/ USA	2014-2015	260	PD	drain	–	–	190	–	–	4
						no drain	–	–	70	–	–	
Mehta et al. (14)	retrospective	USA	2005-2012	709	PD	drain	60	130/121	251	162/89	27.3	8
						no drain	62.5	222/236	458	289/169	26.6	
Paulus et al, (32)	retrospective	USA	1997-2011	69	DP	drain	52 (44-66)	–	39	27/11	–	7
						no drain	58 (52-68)	–	30	25/5	–	
Seykora et al. (16)	retrospective	USA	2014-2017	5581	DP	drain	61.2±13.9	2111/2597	4708	–	–	7
						no drain	60.8±14.9	337/536	873	–	–	
Xourafas el al (38)	retrospective	USA	2014-2016	6730	PD	drain	–	3173/2705	5878	4682/1196	–	8
						no drain	–	426/426	852	704/148	–	
RCT studies												
Van Buren et al, (17)	RCT	USA	2011-2012	137	PD	drain	62.1 (11.7)	37/31	68	45/23	27.8±7.7	–
						no drain	64.3 (12.6)	38/31	69	50/19	27.6±6.1	
McMillan et al, (31)	RCT	Italy	2011-2012	137	PD	drain	62.1 (11.7)	37/31	68	45/23	27.8±7.7	–
						no drain	64.3 (12.6)	38/31	69	50/19	27.6±6.1	
Witzigmann et al. (11)	RCT	Germany	2007-2015	395	PD	drain	64.3 (11.3)	13/72	202	135/67	25.2±4.2	–
						no drain	62.5 (12.2)	126/67	193	115/78	24.9±4.3	
Van Buren et al (18)	RCT	USA	2011-2016	344	DP	drain	61 (49-73)	72/102	174	85/86	28.6 (25.2-33.4)	–
						no drain	60 (47-73}	67/103	170	89/ 84	27.7 (23.6-32.6)	

RCT, random control trial.

### Outcomes

#### Mortality

**Figure 3:**
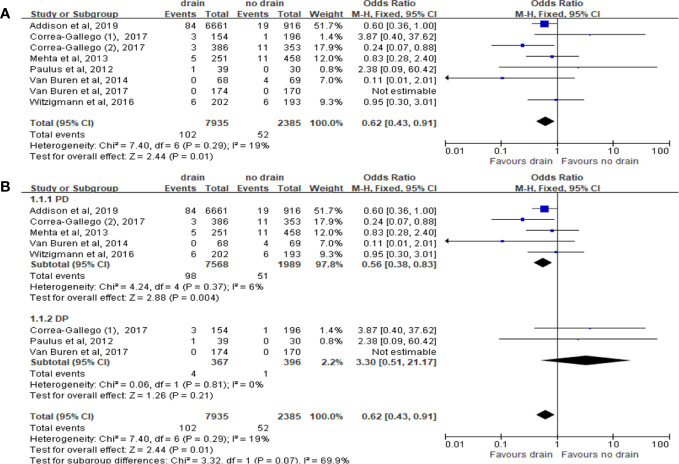
Forest plot of the comparison of 30d-mortality in drain versus no-drain groups after pancreatic resection. **(A)** Comparison in overall; **(B)** comparison in PD and DP subgroups, respectively.

We included 7 studies with total 10,320 patients to estimate the effect on 30 days mortality. Mortality was lower in the drain group than in the no-drain group (OR 0.62; 95%CI 0.43-0.91, P= 0.01) (**Figure 3A**). In PD subgroup, the pooled analysis showed the same tendency as in the overall (OR 0.56; 95%CI 0.38-0.83; P= 0.004) (**Figure 3B**), but there was no significant difference in DP subgroup (P=0.21). For subgroups by FRS, only two studies provided the data (33, 38). There was no significant difference between drain group and no-drain group, either in the Low-risk subgroup (P=0.76) or the High-risk subgroup (P=0.29).

#### Morbidity

**Figure 4:**
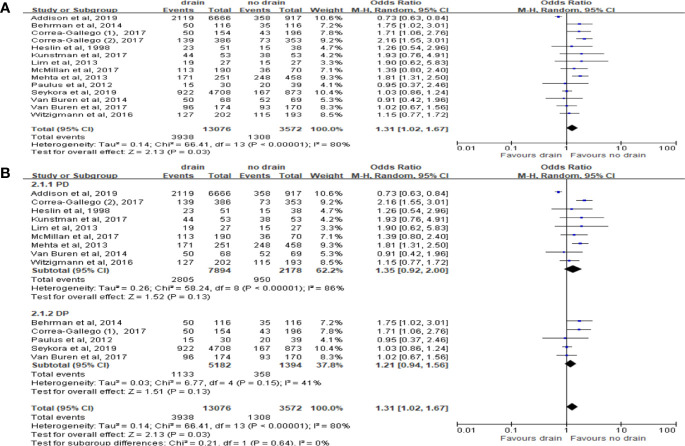
Forest plot of the comparison of morbidity in drain versus no-drain groups after pancreatic resection. **(A)** Comparison in overall; **(B)** comparisons in PD and DP subgroups, respectively.

Total 13 studies were included with 16,648 patients. The results in random effect model showed that morbidity was higher in the drain group (OR 1.31; 95%CI 1.02-1.67; P=0.03) (**Figure 4A**), in the presence of heterogeneity (I^2 =^ 80%). However, there was no significant difference in both PD and DP subgroups (P=0.13) (**Figure 4B**). In contrast to subgroups by the types of surgery, two studies (33, 38) showed that the drain group had a higher morbidity rate than the no-drain group in low risk subgroup (OR 1.23, 95%CI 1.03-1.48, P=0.02), but in High-risk subgroup, the morbidity rate in the drain group was lower (OR 0.76, 95%CI 0.59-0.98, P=0.03).

#### CR-POPF

**Figure 5:**
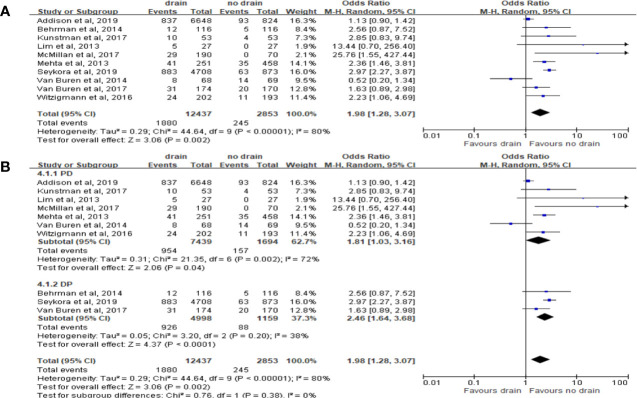
Forest plot of the comparison of the rate of CR-POPF in drain versus no-drain groups after pancreatic resection. **(A)** Comparison in overall; **(B)** comparison in PD and DP subgroups, respectively.

**Figure 6:**
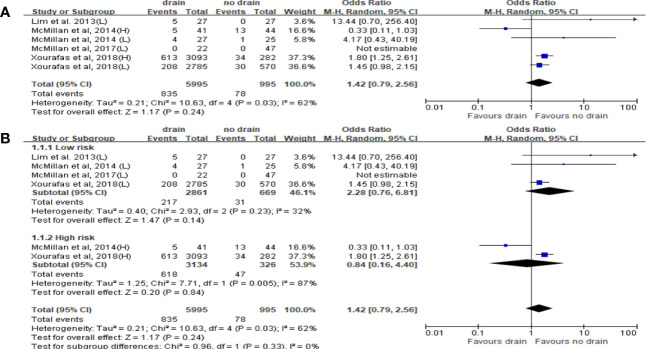
Forest plot of the comparison of the rate of CR-POPF in drain versus no-drain groups after pancreatic resection. **(A)** Comparison in overall; **(B)** comparison in Low risk and High risk subgroups, respectively.

Ten studies reported the data of CR-POPF with total 15,290 patients. The rate of CR-POPF was significantly higher in the drain group than in the no-drain group in the pooled analysis (OR 1.98, 95%CI 1.06-4.69; P=0.002) (**Figure 5A**). The results of PD and DP subgroups were the same as the rate of CR-POPF in overall pool (PD, DP; OR 1.81, 2.46; 95%CI 1.03-3.16, 1.64-3.68; P<0.00001, P=0.0002; respectively) (**Figure 5B**). Four studies reported FRS before placing drainage tube after PD (31, 33, 36, 38). The results based on these studies showed no significant difference in the rate of CR-POPF between two groups in overall (P=0.24) or High-risk subgroup (P=0.84), but the rate of CR-POPF was higher in the drain group in Low risk subgroup (OR 1.50, 95%CI 1.09-2.36, P=0.02) (**Figure 6**).

#### Bile Leak

**Figure 7:**
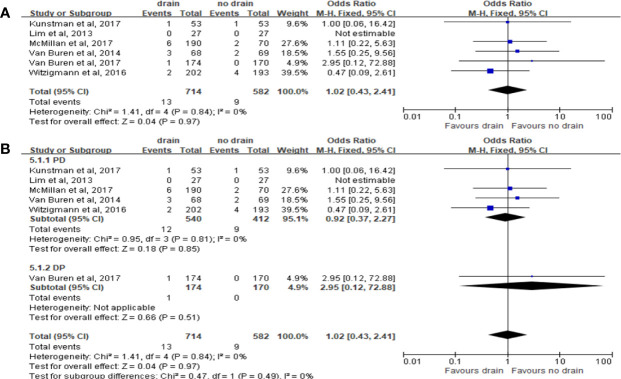
Forest plot of the comparison of the rate of biliary fistula in drain versus no-drain groups after pancreatic resection. **(A)** Comparison in overall; **(B)** comparison in PD and DP subgroups, respectively.

Six studies reported the incidence of bile leak. The pooled meta-analysis showed no significant difference between the two groups (OR 1.02, 95%CI 0.43-2.41, P=0.97) (**Figure 7A**), both in PD (OR 0.92, 95%CI 0.37-2.27, P=0.85) and DP subgroups (OR 2.95, 95%CI 0.12-72.88, P=0.51) (**Figure 7B**).

#### DGE

**Figure 8:**
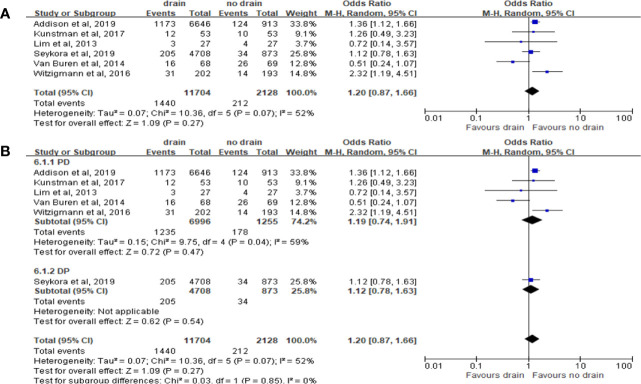
Forest plot of the comparison of the rate of delayed gastric emptying in drain versus no-drain groups after pancreatic resection. **(A)** Comparison in overall; **(B)** comparison in PD and DP subgroups, respectively.

Pooled analysis with 5 studies on DGE showed no significant correlation with the drain or no-drain groups (OR 1.20, 95%CI 0.87-1.66, P=0.27) (**Figure 8A**). Similar results were shown in the subgroup analysis for PD and DP (P=0.47 and P=0.54; respectively) (**Figure 8B**). However, for subgroup analysis of FRS, the incidence of DGE in drain group was significantly higher in the Low-risk subgroup (OR 1.49, 95%CI 1.11-1.99, P=0.007), but not in the High-risk subgroup (P=0.51).

#### Post-Operation Hemorrhage

**Figure 9:**
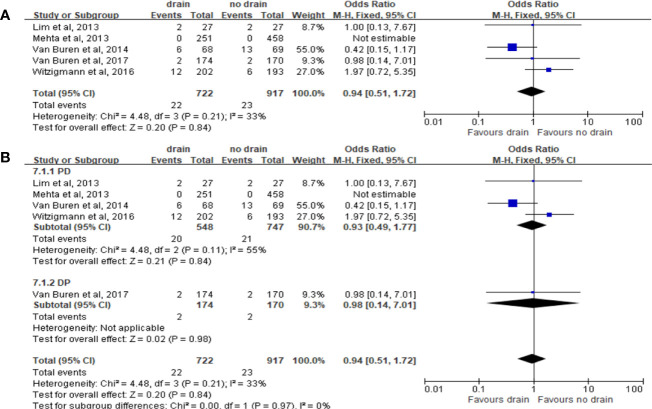
Forest plot of the comparison of the rate of hemorrhage in drain versus no-drain groups after pancreatic resection. **(A)** Comparison in overall; **(B)** comparison in PD and DP subgroups, respectively.

Pooled analysis of 5 studies on the incidence of post-operation hemorrhage showed no significant difference in the rate of hemorrhage in the drain and no-drain groups (OR 0.94, 95%CI 0.51-1.72, P=0.84) (**Figure 9A**), as well as in subgroups (PD, DP; P=0.84, P=0.98; respectively) (**Figure 9B**). However, the rate of hemorrhage in drain group was higher than in the no-drain group in both Low-risk (OR 2.11, 95%CI 1.61-2.77, P<0.00001) and High-risk subgroups (OR 1.78, 95%CI 1.18-2.70, P=0.006), based on the data of two studies (33, 38).

#### Intra-Abdominal Abscess

**Figure 10:**
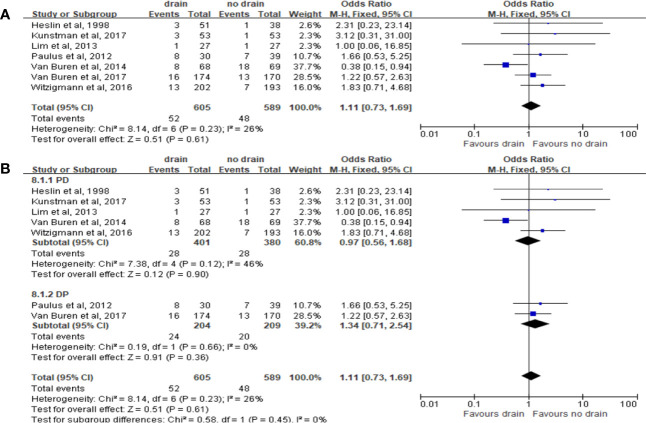
Forest plot of the comparison of the rate of intra-abdominal abscess in drain versus no-drain groups after pancreatic resection. **(A)** Comparison in overall; **(B)** comparison in PD and DP subgroups, respectively.

Pooled analysis of 7 studies on the incidence of intra-abdominal abscess showed no significant difference in the drain and no-drain groups (OR 1.11, 95%CI 0.73-1.69, P=0.61) (**Figure 10A**), both in PD (P=0.90) and DP (P=0.36) subgroups (**Figure 10B**). Only one study provided the relevant data about subgroups of FRS. Lim et al. (33) observed no significant difference between two groups for patients at low risk of pancreatic fistula (4% *vs*. 0%, P=0.31).

#### Wound Infection

**Figure 11:**
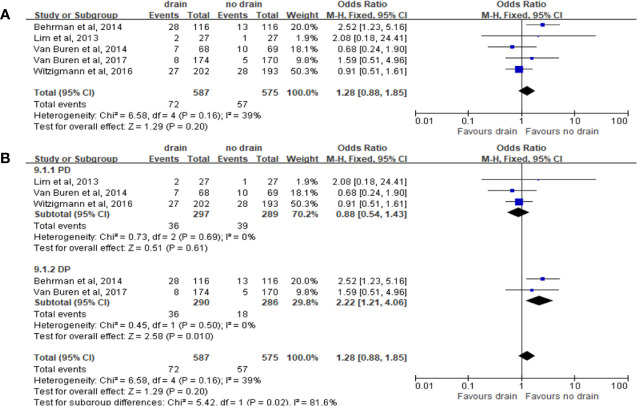
Forest plot of the comparison of the rate of wound infection in drain versus no-drain groups after pancreatic resection. **(A)** Comparison in overall; **(B)** comparison in PD and DP subgroups, respectively.

The results of pooled analysis of 5 studies on the rate of wound infection showed no significant difference between the drain and no-drain groups on the rate of wound infection (OR 1.28, 95%CI 0.88-1.85, P=0.20) (**Figure 11A**). The same results were shown in PD subgroup (OR 0.88, 95%CI 0.54-1.43, P=0.61) (**Figure 11B**). However, in DP subgroup the rate of wound infection was significant higher in the drain group than in the no-drain group (OR 2.22, 95%CI 1.21-4.06, P=0.01) (**Figure 11B**). For subgroups of FRS, the rate of wound infection showed no significant difference both in Low-risk subgroup (P=0.10) and High-risk subgroup (P=0.44).

#### IR

**Figure 12:**
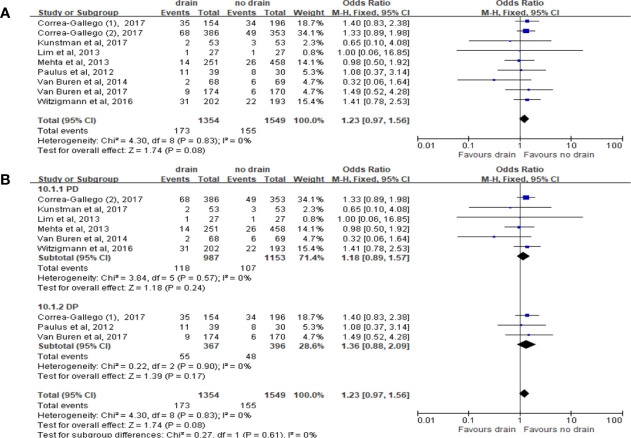
Forest plot of the comparison of the rate of intervened radiology in drain versus no-drain groups after pancreatic resection. **(A)** Comparison in overall; **(B)** comparison in PD and DP subgroups, respectively.

Pooled analysis of 8 studies on the incidence of IR with 2,903 patients showed no significant difference in the drain and no-drain groups (OR 1.23, 95%CI 0.97-1.56, P=0.08) (**Figure 12A**), both in PD (P=0.24) and DP (P=0.17) subgroups (**Figure 12B**). The data about subgroups of FRS cannot be accessed from the studies included.

#### Reoperation

**Figure 13:**
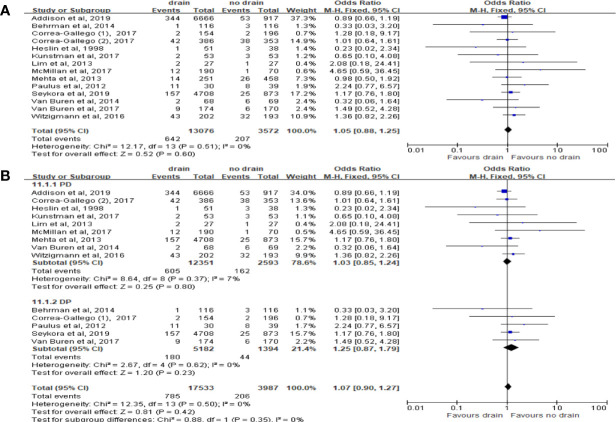
Forest plot of the comparison of the rate of reoperation in drain versus no-drain groups after pancreatic resection. **(A)** Comparison in overall; **(B)** comparison in PD and DP subgroups, respectively.

Pooled analysis of 13 studies on reoperation showed no significant difference between the drain and no-drain groups, either in the overall (OR 1.05, 95%CI 0.88-1.25, P=0.60) or in subgroups of surgery (PD, DP; P=0.80, P=0.23; respectively) (**Figures 13A, B**). According to the studies of Lim et al. (33) and Xourafas et al. (38), the rate of reoperation in the drain group was significantly lower than in the no-drain group in High-risk subgroup (OR 0.57, 95%CI 0.37-0.88, P=0.01), but not in Low-risk subgroup (P=0.77).

#### Readmission

**Figure 14:**
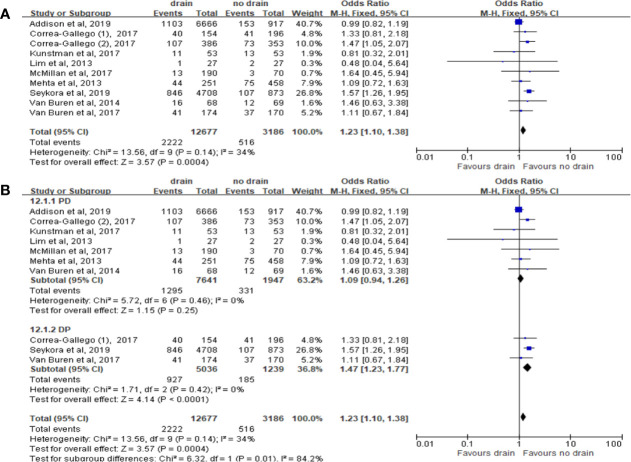
Forest plot of the comparison of the rate of readmission in drain versus no-drain groups after pancreatic resection. **(A)** Comparison in overall; **(B)** comparison in PD and DP subgroups, respectively.

Pooled analysis of ten studies on the incidence of readmission showed that the drain group had significantly higher rate than the no-drain group (OR 1.23, 95%CI 1.10-1.38, P=0.0004) (**Figure 14A**). Similar result was observed in DP subgroup (OR 1.47, 95%CI 1.23-1.77, P<0.0001) but not in PD subgroup (P=0.25) (**Figure 14B**). We found no difference between two groups in Low-risk subgroup (P=0.77) and High-risk subgroup (P=0.22).

### Sensitive Analyses and Publication Bias

Sensitivity analyses were performed by excluding individual study and the alterations of the results on most outcomes were not significant, indicating that our results were stable except for DGE and readmission. Four outcomes including morbidity, CR-POPF, reoperation and readmission were analyzed to assess publication bias (**Supplementary Figure 1**). The funnel plots were presented symmetrically, indicating no publication bias. Furthermore, we performed Begg’s (39) and Egger’s (40) test to confirm that our results were convincing.

## Discussion

In this updated meta-analysis, we showed that there were significant differences between the drain group and the no-drain group in terms of mortality, morbidity, CR-POPF and readmission for all patients undergoing pancreatic resection. Subgroup analysis indicated that mortality of the drain group was lower in PD, but not in DP subgroup. Although the overall morbidity was significantly higher in the drain group, no difference was found in both PD and DP subgroups. For PD, the morbidity in the drain group was higher in Low-risk subgroup, but was lower in High-risk subgroup. The rate of CR-POPF in the drain group was higher in both PD and DP subgroups, as well as in Low-risk subgroup. Drain group had significantly higher rate of readmission than the no-drain group, as well as in DP, but not in PD subgroup. In addition, we found there were no significant differences in bile leak, hemorrhage, DGE, intra-abdominal abscess, and IR in either overall or each subgroup.

Drainage has been a traditional method for the early observation and relief of the clinical relevance of fistula, thereby preventing or reducing postoperative hemorrhage and abscess (9, 23). However, routinely placed drainage during abdominal surgery may be unnecessary or even harmful to some patients, due to the possibility of increasing complications (41–45). In this study, we found that for PD, the mortality in the drain group was lower than in the no-drain group, consistent with the results of Correa-Gallego et al. and Van Buren et al. (15, 17). However, no significant difference was found on morbidity between drain group and no-drain group, and rate of CR-POPF was significantly higher in the drain group than in the no-drain group. POPF is a common and major factor related to the morbidity and mortality in patients undergoing pancreatic resection, and is one of the most concerned complications, regardless of the type of surgical procedure (46, 47). Therefore, CR-POPF is paid more attention after surgery and is a key index in clinical practice. Our results and some nRCTs revealed that drainage might increase the rate of CR-POPF after both PD and DP (11, 14, 36), indicating that drainage should not be referred as a routine measure to reduce the occurrence of complications.

McMillan et al. (36) divided patients with PD into two groups according to FRS: negligible/low risk and moderate/high risk, and showed that drainage can be safely omitted for one-quarter PD patients. However, in this study drainage was routinely used for all patients with moderate/high risk. Lim et al. (33) observed in 27 consecutive PD patients at low risk of pancreatic fistula and found that abdominal drainage should not be placed for PD patients at low risk. After integrating and analyzing all the related studies, our results showed that prophylactic intra-abdominal drainage could significantly increase the rate of CR-POPF in Low-risk subgroup, but not in High-risk subgroup. For Low-risk subgroup, the rates of hemorrhage, DGE and morbidity were higher. For High-risk subgroup, the rate of hemorrhage was higher while the rates of reoperation and morbidity were lower in the drain group. Other outcomes showed no significant differences between Low-risk and High-risk subgroups. Therefore, our results confirmed the conclusion that for patients with low fistula risk, prophylactic drainage might be associated with even higher morbidity after PD. In contrast, patients with high fistula risk might benefit from the drain placement.

For DP, several studies have been conducted to evaluate the effect of routine drainage placement. Paulus et al. (32) found that the drain did not decrease morbidity or the need for further intervention. Furthermore, it is of little significance in the diagnosis of complications. Van Buren et al. (18) showed that there was no difference in the rate of POPF between drain and no drain groups. Moreover, Behrman et al. (48) concluded that placement of drains following elective distal pancreatectomy was associated with a higher overall morbidity and pancreatic fistulas. Mangieri et al. (49) found a significant increase in the rates of readmission with the placement of surgical drain after DP. In the current study, we found that there was no significant difference between the drain group and the no-drain group in terms of mortality and morbidity, as well as bile leak, hemorrhage, DGE, intra-abdominal abscess, IR and reoperation. However, the rates of CR-POPF, wound infection and readmission were all significantly higher in the drain group, which were partially consistent with the results of previous studies. Hence, the drainage might not be advocated as a routine method in DP procedure. However, the final conclusions can still not be drawn due to lack of RCT specifically for DP so far. Furthermore, the lack of FRS specifically for DP also limited the conclusion for this procedure. Recently, Ecker et al. (50) tried to identify a clinical FRS following DP, but their result failed to predict the rate of CR-POPF reliably.

There are some limitations in this meta-analysis. First, most of included studies were nRCT and only four RCTs were included, which may reduce the level of evidence. Second, some results were unstable after omitting some studies on DGE and readmission. Third, the use of drains was no standardized, for example, the types of drains and how long they were kept in site. Fourth, the definitions of some outcomes in some studies were not universal, especially for the classification of POPF, which might influence the proper comparison of these complications. Fifth, due to the lack of data in the literatures, we could not exclude potential confounding factors, such as pancreatic textures, pancreatic duct caliber, and body mass index, which are correlated with the incidences of POPF and other complications after pancreatic surgery (51).

In conclusion, intraperitoneal drainage may benefit some patients undergoing PD, especially those with high pancreatic fistula risk. For DP, current evidences suggest that routine drainage might not benefit patients, but no clear conclusions can be drawn because of the study limitations. Further studies are demanded on this topic.

## Data Availability Statement

The original contributions presented in the study are included in the article/**Supplementary Material**. Further inquiries can be directed to the corresponding authors.

## Author Contributions

XT is the guarantor. XL, XT, KC, and YY contributed to the conception of the study. The search strategy was developed by all authors and was performed by XL and KC, who also independently screened the potential studies, extracted data from included studies, and assessed the risk of bias. XL and XT conducted and finished the data synthesis. XC and GL arbitrated in cases of disagreement and ensured no errors occur during the study. All authors contributed to the article and approved the submitted version.

## Funding

This study was supported by the Natural Science Foundation of China (81672353, 81871954) and Beijing Natural Science Foundation (No.7212111).

## Conflict of Interest

The authors declare that the research was conducted in the absence of any commercial or financial relationships that could be construed as a potential conflict of interest.
