# Supplementary figures and images for: Prophylactic Intra-Peritoneal Drainage After Pancreatic Resection: An Updated Meta-Analysis
Source: Front Oncol. 2021 May 20;11:658829. doi: 10.3389/fonc.2021.658829 (PMC8172774; doi:10.3389/fonc.2021.658829)

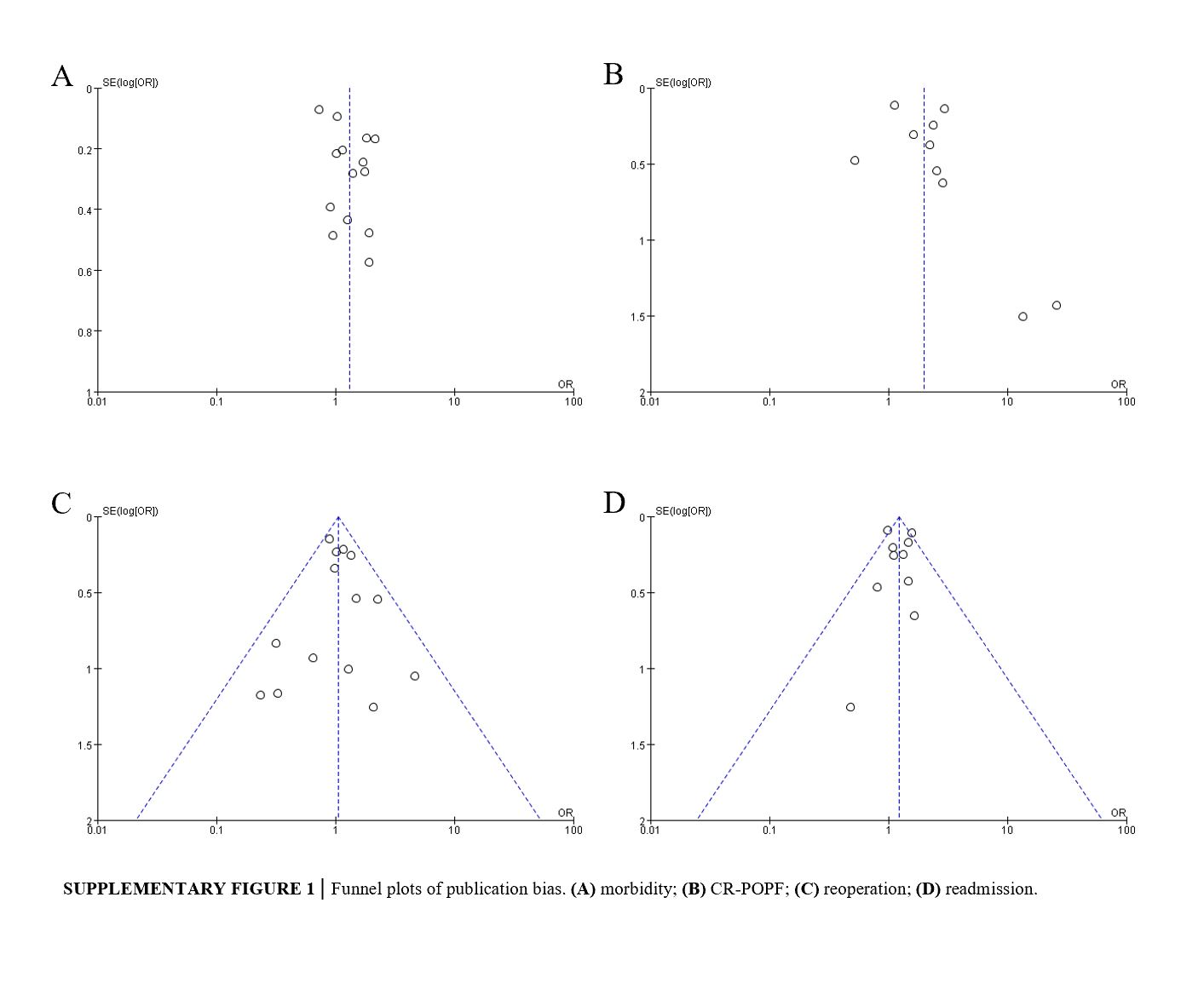

Supplement: Supplementary file 1 [file Image_1.jpg]
